# Global climatology of submesoscale restratification using machine learning

**DOI:** 10.1038/s41598-026-41929-x

**Published:** 2026-03-20

**Authors:** Leyu Yao, John R. Taylor

**Affiliations:** Department of Applied Mathematics and Theoretical Physics, University of Cambridge, Wilberforce Road, Cambridge, CB3 0WA UK

**Keywords:** Submesoscale eddy, Profile classification model (PCM), Argo float, Climate sciences, Ocean sciences

## Abstract

Submesoscale eddies are important in setting the stratification in the ocean surface mixed layer and transporting energy between large and small scale motions. However, the study of submesoscale on a global scale has been hindered by a shortage of global, long-term datasets. To meet this need, we apply an unsupervised machine learning method adapted from the profile classification model (PCM) to density profiles collected by Argo floats over the global ocean from 2000-2021, producing the first global observational climatology of submesoscale restratification. The method classifies individual vertical profiles based on the shape of the density profile in the ocean surface mixed layer. The fraction of profiles that exhibit a shape characteristic of submesoscale is referred to as the submesoscale restratification (SR) index. The SR index peaks in spring in both hemispheres and lags the maxima of mixed layer depth by one month, suggesting that submesoscale eddies play an important role in restratifying the mixed layer. Hotspots of SR index can be found in the Norwegian Sea and the Drake Passage in spring. This method enables the study of the spatial and temporal distributions of submesoscale restratification on a global scale.

## Introduction

Submesoscales are dynamic ocean features with horizontal scales from 0.1-10 km that are characterised by a Rossby number of $Ro=\zeta /f\sim \mathcal {O}(1)$, where $\zeta$ is the surface vertical vorticity and *f* the Coriolis frequency^1–3^. Submesoscales are ubiquitous features of the upper ocean where they have a strong influence on the structure of the water column, vertical transport, and biogeochemistry. Vertical motion associated with submesoscales has a strong direct impact on biogeochemical cycles^4,5^ and heat transport in the upper ocean^6,7^.

Submesoscales in the upper ocean generally form through a variety of fluid dynamical instabilities in regions with horizontal density gradients. In particular, mixed layer instability (MLI) is an ageostrophic baroclinic instability that generates 1-10 km eddies^8,9^, while symmetric instability generates coherent velocity bands that are typically aligned with sloping isopycnals and nearly independent of the along-front direction. Both instabilities draw energy from the potential energy associated with sloping density contours and the associated velocity and result in an increase in the stable density stratification of the upper ocean^1^. This increase in stratification reduces the depth of the ocean surface mixed layer, with potentially important impacts on climate model forecasts^10,11^.

Although understanding of submesoscale dynamics has been greatly advanced in recent years with the development of satellite remote sensing, autonomous underwater vehicles, and high-resolution numerical models, direct observations of submesoscale eddies are still largely limited to high-resolution targeted field surveys with limited domain size and duration, and global models are just reaching the resolution to resolve submesoscale motions^7,12^. The shortage of global, long-term observations and datasets of submesoscale eddies hinders the study of their distribution, seasonality, and inter-annual variations on a global scale. In this paper, we show that a machine learning method that infers the influence of submesoscale restratification from existing global multi-year datasets of the ocean has the potential to provide insight into the influence of submesoscales on the density structure of the upper ocean.

The unsupervised machine learning method we use in this paper was introduced and tested using submesoscale-resolving simulations in^13^. The method is based on the profile classification model (PCM) first proposed in^14^ and used to analyse oceanographic vertical profiles in recent years^14–19^. In particular, the PCM has been effective in identifying regimes in the ocean with similar vertical structures when applied to data from ocean floats, especially those from the Argo Program^14,17,18^. The PCM classifies ocean vertical profiles by decomposing the multi-dimensional probability density function (PDF) of a dataset into a weighted sum of Gaussian distributions^14^. Yao et al.^13^ adapted the PCM by adding a scaling procedure to the dataset that extracts and rescales the portion of vertical density profiles in the ocean surface mixed layer. With this modification, the method classifies individual profiles purely based on the *shape* of the vertical density profile.

In^13^, the adapted PCM was applied to two model-based datasets, one of which was extracted from the high-resolution, idealised domain large-eddy simulation (LES) from^20^ and the other from a regional model of the Southern Ocean from^21^. With the added scaling process, the adapted PCM was able to identify the signature of submesoscale eddies in individual vertical density profiles without any information on the velocity, location of the profiles, or horizontal density gradients, providing a method to study submesoscales using global datasets^13^. Previous modelling work has found that submesoscales can persist under strong forcing without restratifying the surface mixed layer^22^. In these cases the adapted PCM might be able to detect the influence of submesoscales. Instead, this method detects evidence for submesoscale restratification based on the shape of the density profile in the mixed layer.

Here we apply this method to a global dataset from Argo floats that contains over 20 years of water column measurements^23^. In particular, we use all the density profiles collected by Argo floats during the night (22:00-6:00) in local time from 2000 to 2021 to avoid the influence of solar heating on the profile shapes during the day (see ‘Discussion’). We define the ‘submesoscale restratification’ (SR) index as the fraction of profiles in a given region during a given period of time that are classified by the adapted PCM method as being influenced by submesoscale restratification. By calculating the SR index from the global ARGO dataset from 2000 to 2021, we produce a global observational climatology of submesoscale restratification which allows the study of submesoscales and their influence on the upper ocean stratification on a global scale.

## Results

### First global observational climatology of submesoscale restratification

**Figure 1:**
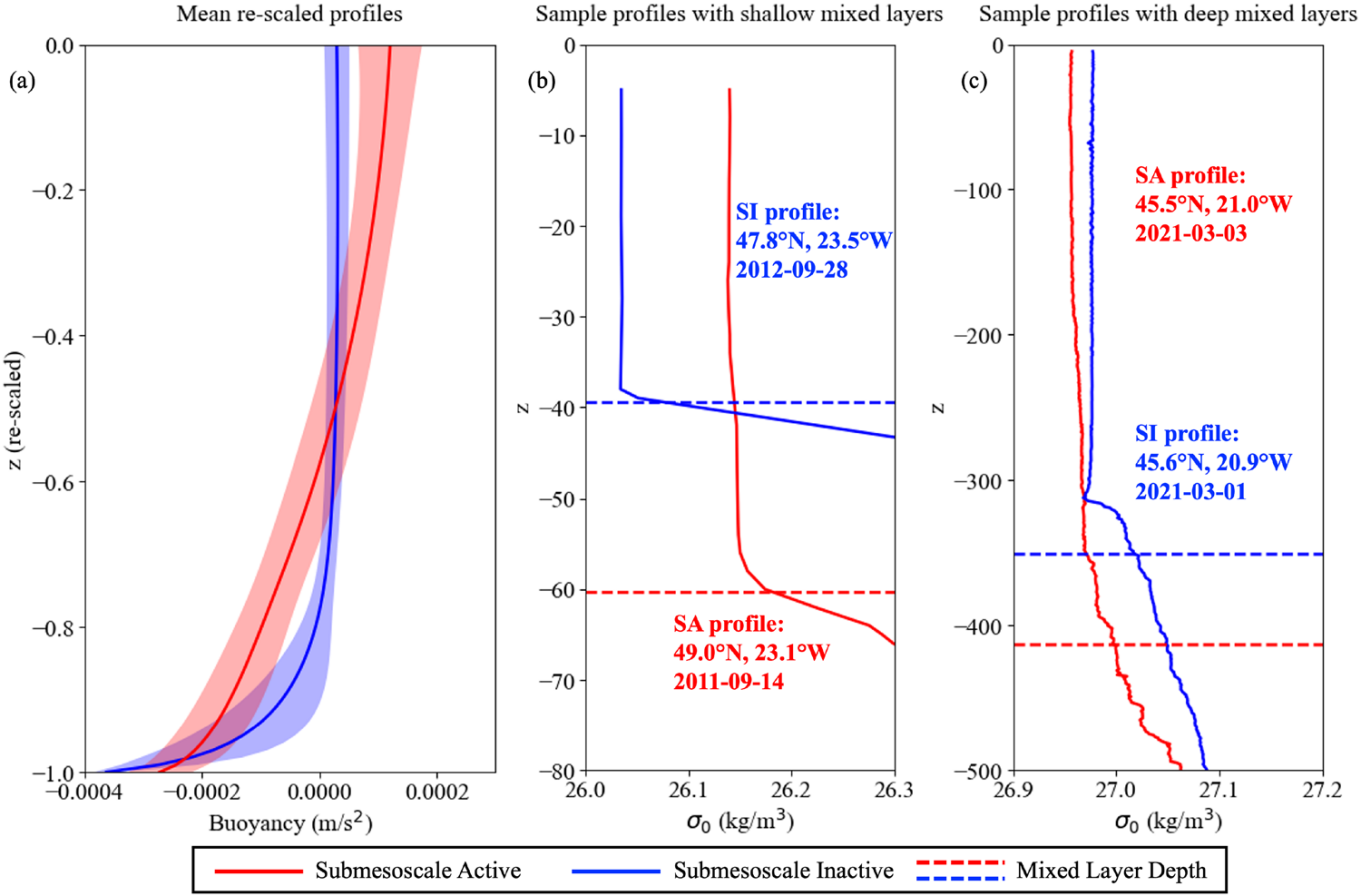
(**a**) Mean rescaled profiles in mixed layer for Submesoscale Active (SA) and Submesoscale Inactive (SI) classes, where solid lines show the mean profiles for each class and shaded areas show one standard deviation ranges, and sample potential density anomaly ($\sigma _0$) profiles with (**b**) shallow mixed layers and (**c**) deep mixed layers, where red profiles are classified as SA, blue profiles are classified as SI, and dashed lines indicate mixed layer depths. All four sample profiles are from the same $5^\circ$ by $5^\circ$ latitude-longitude box used for analysis in Fig. 3.

When applied to the Argo dataset, the adapted PCM method from^13^ produces two distinct classes of density profiles with very different shapes (see ‘Methods’). In one class (blue profiles in Fig. 1), the density is relatively constant in the mixed layer and increases abruptly at the base of the mixed layer. In the other class (red profiles in Fig. 1), the density increases more gradually from the ocean surface to the base of the mixed layer, with weak but non-zero stratification in the mixed layer. This shape is consistent with the signature of submesoscale eddies formed through mixed layer instability (MLI) which increases the stratification in the mixed layer while decreasing the stratification in the upper thermocline^24^. In^13^, classes with very similar density profile shapes were found when the adapted PCM method was applied to model-based datasets, and profiles with stratification in the mixed layer were very likely to coincide with a submesoscale eddy. In particular, when the method was applied to a realistic high resolution model of the Drake Passage region of the Southern Ocean^21^, the majority (74.57%) of profiles with large local vorticity $|\zeta |>5\times 10^{-5} s^{-1}$ had density profiles that were classified as belonging to a submesoscale eddy.

### Seasonality of submesoscale restratification

**Figure 2:**
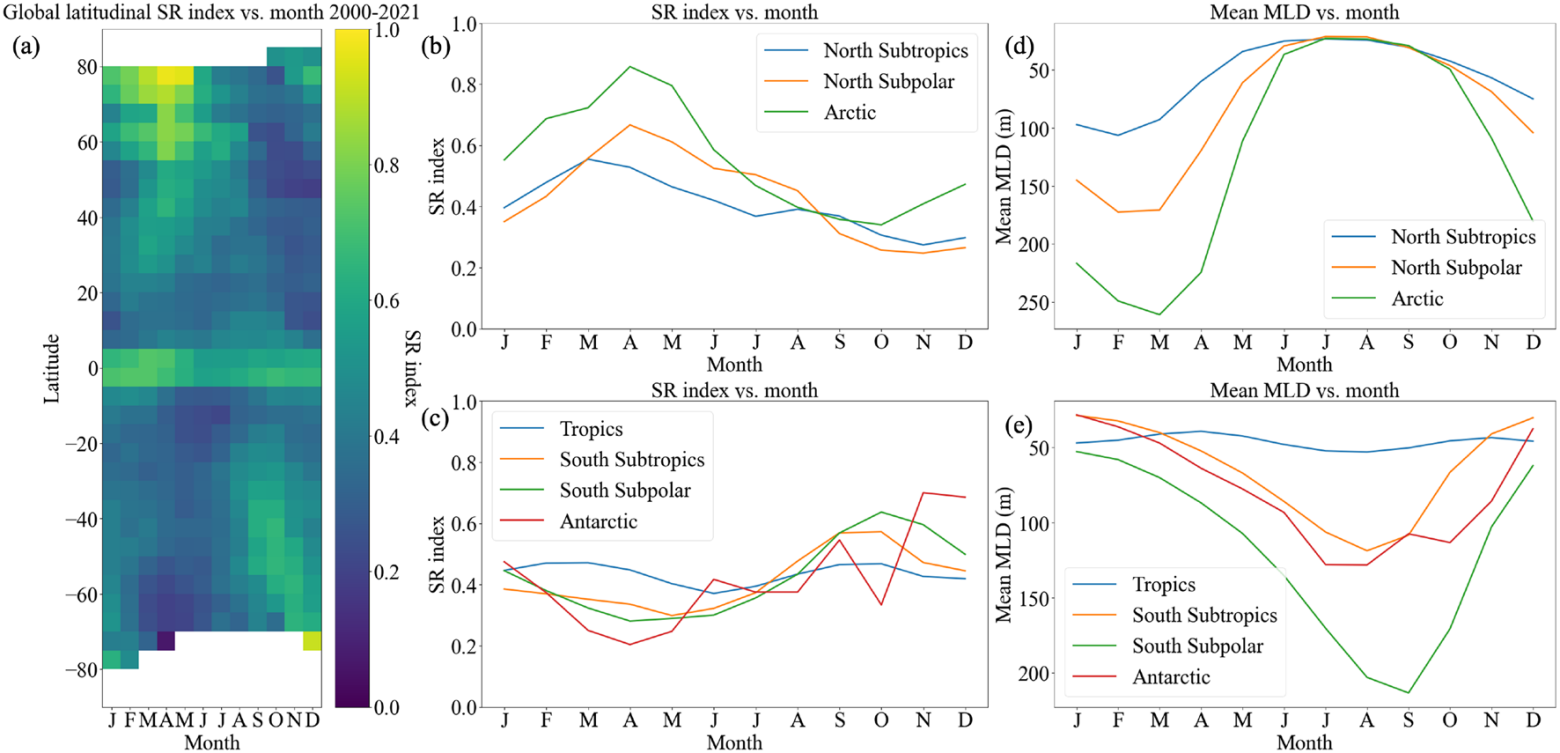
(**a**) Heatmap of global latitudinal SR index, averaged for all longitudes and for each month from 2000-2021, SR index for each month in (**b**) North Subtropics ($23.5^{\circ }$N-$40^{\circ }$N), North Subpolar ($40^{\circ }$N-$66.5^{\circ }$N), Arctic ($66.5^{\circ }$N-$90^{\circ }$N) regions, (**c**) Tropics ($23.5^{\circ }$S-$23.5^{\circ }$N), South Subtropics ($40^{\circ }$S-$23.5^{\circ }$S), South Subpolar ($66.5^{\circ }$S-$40^{\circ }$S), Antarctic ($90^{\circ }$S-$66.5^{\circ }$S) regions for years 2000-2021, and mean MLD for each month in (**d**) North Subtropics, North Subpolar, Arctic regions, and (**e**) Tropics, South Subtropics, South Subpolar, Antarctic regions for years 2000-2021.

When averaged over all longitudes, the SR index exhibits a clear seasonal signal (Fig. 2) with a maximum in spring in both hemispheres. The peak in the SR index occurs later in the year at high latitudes compared to low and mid-latitudes. For context, the maximum mixed layer depth (MLD) occurs in March and September in the Northern and Southern Hemisphere, respectively, and the surface buoyancy forcing reaches maxima around December and June, respectively (Supplementary Fig. 2). The maximum SR index lags the peaks in MLD and surface buoyancy forcing by 1-3 months, respectively. This is consistent with modelling work by Dong et al.^12^, who found that the peak in submesoscale energy lags the maximum MLD by about one month. Interestingly, extrema in the SR index coincide with periods when the *rate of change* in MLD is largest. This supports previous work suggesting that submesoscales play an important role in mixed layer restratification in the spring^25–27^. The largest values of SR index also tend to occur in regions with deep winter mixed layers (Supplementary Fig. 1) which is consistent with previous studies which have found that energetic submesoscales generally coincide with deep mixed layers^12,28,29^.

### Global distribution of submesoscale hotspots

**Figure 3:**
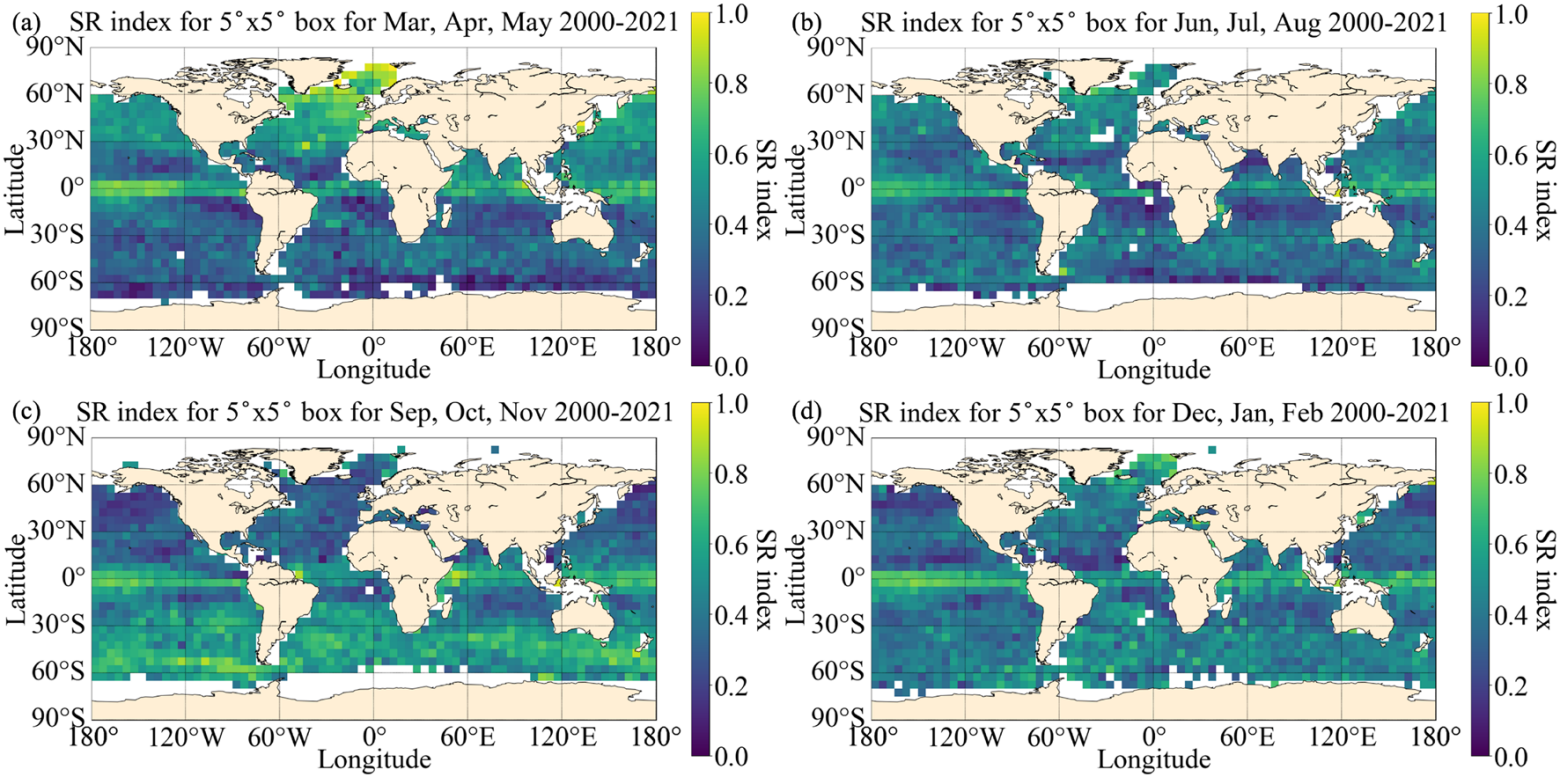
SR index calculated using all profiles collected at night (22:00-6:00) local time in $5^\circ$ by $5^\circ$ latitude-longitude boxes for (**a**) March, April, May, (**b**) June, July, August, (**c**) September, October, November, and (**d**) December, January, February for 2000–2021.

The SR climatology sheds light on the global distribution of submesoscale hotspots (Fig. 3). In springtime in the Southern Hemisphere, the SR index is large along the Antarctic Circumpolar Current (ACC) in the Southern Ocean, particularly in the Drake Passage region. Another hotspot of the SR index occurs in the North Atlantic in spring in the Northern Hemisphere, particularly in the Norwegian Sea. It is also noted that the SR index is relatively high near the equator in all seasons, the cause of which is unclear. This region will be discussed further below.

### Restratification ratio

Other measures have been proposed to quantify the ability of submesoscales to restratify the surface mixed layer. Stratification in the upper ocean is eroded by wind and surface buoyancy forcing, which can be quantified by the combined buoyancy flux due to destabilising heat and freshwater air-sea fluxes and the contribution from Ekman transport across fronts^30–32^. On the other hand, baroclinic mixed layer instability, which generates submesoscale eddies, restratifies the mixed layer through a vertical buoyancy flux^9^. Combining the competing effects, Mahadevan et al.^25^ defined the restratification ratio, *R*, as the ratio of the surface buoyancy flux to the buoyancy flux associated with parametrised submesoscale restratification.

Here, we calculate the restratification ratio using global observational and model data for the years 2020 and 2021 (see ‘Methods’). When all points are combined, there is a clear correlation between SR index and the restratification ratio *R*. In particular, points with $R>10$ have an average SR index of 0.2887, while points with $R<0$ have an average SR index of 0.4572 (Supplementary Fig. 3). As expected, large values of the restratification ratio (e.g., $R>10$) indicate that mixing generated by surface forcing outpaces submesoscale restratification, resulting in a lower average SR index. The same correlation between SR index and the restratification ratio can be seen spatially with clear seasonal shifts. The SR index is low in the Southern Ocean from March-July. During these months (austral autumn and winter), high values of the restratification ratio are prevalent in the same region (Supplementary Fig. 4a,b). Similarly, from September-February low SR index and high restratification ratio occur in the Northern Atlantic and North Pacific Oceans (Supplementary Fig. 4c,d).

## Discussion

Although the Southern Ocean and the general path of the Antarctic Circumpolar Current has a large SR index in spring, other frontal regions with large horizontal density gradients including western boundary currents and major coastal upwelling systems (e.g. the Gulf Stream and the California Current regions) do not exhibit clearly elevated SR index. A map of the horizontal density gradient from a global state estimate is provided in Supplementary Fig. 8 for comparison with the SR index. While some regions with high horizontal density gradient correspond to regions with high SR index, there is not a clear 1:1 correspondence. This is surprising since submesoscales are known to develop in frontal systems with large horizontal density gradients. Western boundary currents which experience de-stabilizing wind forcing and strong surface heat loss have large values of the restratification ratio (Supplementary Fig. 4) which might explain the lack of a peak in the SR index in these frontal systems. Callies and Ferrari^22^ noted that submesoscale eddies can persist during periods of strong forcing (with large restratification ratio), without having a strong influence on the vertical density profile. Hence, the SR index might be an underestimate of the level of submesoscale activity in these regions.

When training the adapted PCM and applying the model to profiles, we only use the density profiles collected by Argo floats during the night (22:00-6:00) in local time to minimise the influence of solar heating on the density profile. Solar radiation heats up the surface of the ocean and can create a thin diurnal warm layer. The diurnal warm layer is most prominent on days of high solar radiation and low wind speed, where the changes in sea surface temperature can reach a few degrees^33–36^. The diurnal warm layer changes the shape of the top of the temperature profile and density profile in the ocean mixed layer, which can be detected by the uppermost several measurements of Argo floats and can affect the classification results produced by the unsupervised machine learning model. Specifically, when the adapted PCM is trained with randomly selected profiles from all available density profiles collected by the Argo floats from all times of day from 2000 to 2021, more daytime (10:00-18:00 local time) profiles are categorised as SA than nighttime (22:00-6:00) profiles in the region between $40^{\circ }$N and $40^{\circ }$S where solar insolation is largest (Supplementary Fig. 5a). This indicates that the diurnal warm layer caused by solar heating produces a bias toward SA near the equatorial region. To avoid this effect, we only use profiles collected at night.

Although we only use profiles collected at night, there is a persistent peak in the SR index near the equator, especially between $5^{\circ }$N and $5^{\circ }$S throughout the year (Fig. 3). The cause of the peak in SR index along the equator is unclear, but there could be several possible explanations. One possible cause is the intense precipitation along the equator brought by the Inter-Tropical Convergence Zone (ITCZ), where surface winds converge and a narrow belt of precipitation occurs^37–39^. Intense rainfall could generate ‘barrier layers’, or regions at the base of the mixed layer where a density jump is large enough to limit mixing from winds and convection but not large enough to trigger the definition of a mixed layer (based on our chosen density difference). Localised sources of freshwater from precipitation or rivers in other regions could similarly generate barrier layers which distort the density profile. It might be possible to isolate barrier layers from submesoscale restratification using additional classes, but this is left for future work. We also note that the horizontal buoyancy gradient and the eddy kinetic energy (EKE) are large in some of the regions near the equator with high SR index (Supplementary Figs. 7 and 8). This suggests that eddy activity and horizontal processes could account for the mixed layer stratification in these regions.

Although there are clear geographical and seasonal trends in the SR index, $97.50\%$ of the regions have SR index between $0.2-0.8$. Globally, the mean SR index is 0.53 for all profiles collected at night during spring months (March, April, May for Northern Hemisphere and September, October, November for Southern Hemisphere) throughout the years 2000-2021. This implies that over half of the profiles collected at night in spring globally bear the signature of submesoscale restratification and that submesoscales are common across large regions of the global ocean and not just major frontal systems or hotspots of eddy kinetic energy. The observation that the maximum in the SR index coincides with the maximum rate of mixed layer shoaling suggests that submesoscales play an important role in seasonal restratification, as suggested by previous work^26,27,29^. Field work targeting hotspots of SR index (e.g., the equatorial regions and Norwegian Sea) would help identify the dynamical processes leading to mixed layer stratification in these regions.

Other potential future work includes applying the adapted PCM to density profiles collected in regions with a known submesoscale eddy field from targeted surveys. It would be particularly useful to investigate regions like western boundary currents and coastal upwelling zones, which are known to exhibit strong submesoscale activity but where the SR index is not notably large. The classification method could also be extended with more classes to distinguish between different types of submesoscale processes, such as symmetric and baroclinic instabilities (see discussion in^13^). It is possible that the use of more classes would allow submesoscales to be detected in regions with strong surface forcing and to identify barrier layers.

## Methods

### Unsupervised machine learning method - adapted PCM

The unsupervised machine learning method we use, the adapted PCM, was introduced in^13^, which was adapted from the profile classification model (PCM) first proposed in^14^. PCM first uses Principal Component Analysis (PCA) to reduce the dimensionality of a dataset, and then applies a Gaussian Mixture Model (GMM) to decompose the probability density function (PDF) of the dataset into a weighted sum of Gaussian distributions^14^. Following the convention for selecting the number of principal components used in the PCA in^14,17,18^, we keep the minimum number of principal components that account for at least $95\%$ of the variance. Since submesoscale eddies mostly affect the mixed layer in the upper ocean, Yao et al.^13^ adapt the PCM by extracting the portion of each profile in the mixed layer and re-scaling and normalising the extracted portion based on the MLD before applying the PCM. The re-scaling procedure interpolates the extracted portion of each profile onto 100 evenly spaced grid points on a dimensionless coordinate from -1 to 0, where -1 indicates the base of mixed layer and 0 indicates the ocean surface. The re-scaled profiles are then normalised by subtracting the vertical mean density from each profile. Following the same practice as in^13^, we use a fixed value of density change with respect to the surface value to determine the MLD for each profile. After comparing multiple commonly used fixed values for determining MLD, we pick the density change $\Delta \rho =0.041$ $\text {kg/m}^3$, which is equivalent to a temperature change of $\Delta T=0.2^\circ$C^40,41^.

### Dataset from Argo floats

The Argo Program has been collecting measurements of water properties in the global ocean using a fleet of autonomous floats since the early 2000s. Argo floats generally drift at 1 km depth in the ocean and around every 10 days, they descend to 2 km depth before returning to the surface and taking measurements on the way back to the ocean surface^23^. While the Argo Program provides data on both hydrographic and biogeochemical properties of the global ocean, here we only use the hydrographic data including absolute salinity, conservative temperature, and potential density anomaly in order to calculate density profiles from Argo floats. We use the Python package ‘argopy’^42^ to filter and fetch all available Argo profiles collected during the night (22:00-6:00) in local time from January 2000 to December 2021 over the whole global ocean. To ensure that all profiles we use contain substantial parts of the mixed layer and include the bases of the mixed layer, we only include Argo profiles with available and quality-controlled measurements at depths between 5 and 900 dbar (approximately 5–900 m) and exclude all profiles with a shallowest depth deeper than 5 dbar or a deepest depth shallower than 900 dbar. After calculating the MLD for each profile, we also discard all profiles where a MLD cannot be defined (e.g. MLD exceeds 900 dbar) and all profiles with fewer than 5 data points in the mixed layer to ensure the shape of the profile in the mixed layer can be well-represented after the re-scaling process. In total, 387,290 profiles pass the filtering and form our dataset for analysis. All profiles in the Argo dataset are then re-scaled and normalised based on their MLDs for the training and testing of the machine learning method.

### Training and application of adapted PCM using Argo dataset

We use the Python package ‘pyXpcm’^43^ to train and test PCM on the rescaled and normalised density profiles in the Argo dataset. For training the PCM, we divide the whole globe into $5^\circ$ by $5^\circ$ latitude-longitude boxes and we randomly select 50 re-scaled and normalised profiles collected at night from 2000-2021 from each box that has sufficient profiles to form the training set, which contains 64,850 profiles. Note that the missing $5^\circ$ by $5^\circ$ latitude-longitude boxes are either land or ocean at very high latitude that has sparse Argo measurements from 2000 to 2021. We train the PCM using the training set with 4 principal components that account for $>95\%$ of the variance. Note that only the randomly selected rescaled and normalised density profiles are fed into the PCM, and the PCM classifies the profiles into groups based purely on the patterns in their shapes without any input truth label or additional information, ensuring the unsupervised nature of the algorithm. The trained model is then applied to all 387,290 profiles in the Argo dataset, producing two classes with distinct profiles in the mixed layer (Fig. 1). The class that is more stratified throughout the mixed layer is assigned to be ‘Submesoscale Active (SA)’, indicating that submesoscales are effective at restratifying the mixed layer, while the other class that is more mixed throughout the mixed layer is assigned ‘Submesoscale Inactive (SI)’.

Note that we use SA and SI to refer to individual profiles, following^13^, but we use the term ‘Submesoscale Restratification’ (SR) index to refer to the fraction of profiles in a given region that are classified as Submesoscale Active. The term Submesoscale Restratification was chosen to emphasise that this measure quantifies the ability of submesoscales to increase the stratification within the mixed layer, while acknowledging that in some regions with strong atmospheric forcing submesoscales might not be capable of generating a stratification signal within the mixed layer. In these regions the SR index could underestimate the presence of submesoscale eddies.

The box size used to generate the SR index was chosen to maximise the number of profiles in each box while still having sufficient resolution to capture global-scale patterns. The number of profiles in the boxes and an estimate of the standard error of the classification result within each box are shown in Supplementary Figs. 9 and 10. The number of profiles is small ($<50$ profiles) in some regions, particularly in the high latitude Southern Ocean, and the results in these regions should be viewed with caution.

### Restratification ratio

Since there is no existing long-term global record of submesoscale activity to compare our classification result with, we calculate the restratification ratio *R*, which is a proxy for the ability of submesoscale eddies to restratify the upper ocean. The restratification ratio measures the competition between the restratification of submesoscale baroclinic instability and the mixing due to convection and down-front winds, and is defined as

$$R = \frac{|f|(B_0+EBF)}{H^2 M^4},$$

where *f* is the Coriolis frequency, $B_0$ is the buoyancy flux due to surface heat and freshwater air-sea fluxes, *EBF* (or Ekman buoyancy flux) is the buoyancy flux due to Ekman transport across fronts, *H* is the mixed layer depth (MLD), and $M^2=\partial \bar{b}/\partial x$ is the large-scale mean buoyancy horizontal gradient. The restratification ratio for the years 2020 and 2021 is calculated using reanalysis products based on observational and model-based data from^44–46^.

In particular, $B_0$ can be estimated as

$$B_0 = -g\left( \frac{\alpha Q_{net}}{\rho C_p} - \beta S_0 F_{net}\right) ,$$

where *g* is the gravitational acceleration, $\alpha$ is the thermal expansion coefficient, $Q_{net}$ is the net surface heat flux, $\rho$ is the density of seawater, $C_p$ is the heat capacity of seawater, $\beta$ is the saline contraction coefficient, $S_0$ is the practical salinity, and $F_{net}$ is the net surface salinity flux. We calculate $Q_{net}$ and $F_{net}$ using ‘mean surface latent heat flux’, ‘mean surface sensible heat flux’, ‘mean surface net shortwave radiation flux’, ‘mean surface net long-wave radiation flux’, ‘mean total precipitation rate’, and ‘mean evaporation rate’ from ERA5 Monthly Averaged Data on Single Levels from 1940 to Present^44^. The other variables $\alpha$, $\rho$, $C_p$, $\beta$, and $S_0$ are calculated with the GSW Oceanographic Toolbox^47^ using ‘sea water potential temperature’ and ‘sea water salinity’ from Global Ocean Physics Reanalysis^46^. We use ‘ocean mixed layer thickness defined by sigma theta’ from^46^ as mixed layer depth *H*.

The Ekman buoyancy flux (EBF) quantifies the mixing induced by the advection of density by Ekman flow when a persistent wind blows down a front (‘down-front wind’), and is defined as

$$EBF = \left( \frac{\boldsymbol{\tau } \times \hat{\textbf{z}}}{\rho f}\right) \cdot \nabla _h b,$$

where $\boldsymbol{\tau }$ is the wind stress, and $\nabla _h b$ is the surface horizontal buoyancy gradient^25,30,31,48^. We calculate surface buoyancy *b* using $\rho$ from the equation above, and obtain $\boldsymbol{\tau }$ using ‘surface downward eastward stress’ and ‘surface downward northward stress’ from the Global Ocean Monthly Mean Sea Surface Wind and Stress from Scatterometer and Model^45^.

## Supplementary Information

Supplementary Information.

## Data Availability

This study has been conducted using E.U. Copernicus Marine Service Information (product 10.48670/moi-00181, 10.48670/moi-00016) and Copernicus Climate Change Service Climate Data Store (product 10.24381/cds.f17050d7).

## Code availability

Code for fetching and processing the Argo data, training the PCM and making all the plots for the paper is available here: https://github.com/Nataliely/Argo-PCM-code.
