# Supplementary material for: Global climatology of submesoscale restratification using machine learning
Source: Sci Rep. 2026 Mar 20;16:14309. doi: 10.1038/s41598-026-41929-x (PMC13144449; doi:10.1038/s41598-026-41929-x)
Supplement: Supplementary file 1 — Supplementary Information. [file 41598_2026_41929_MOESM1_ESM.pdf]

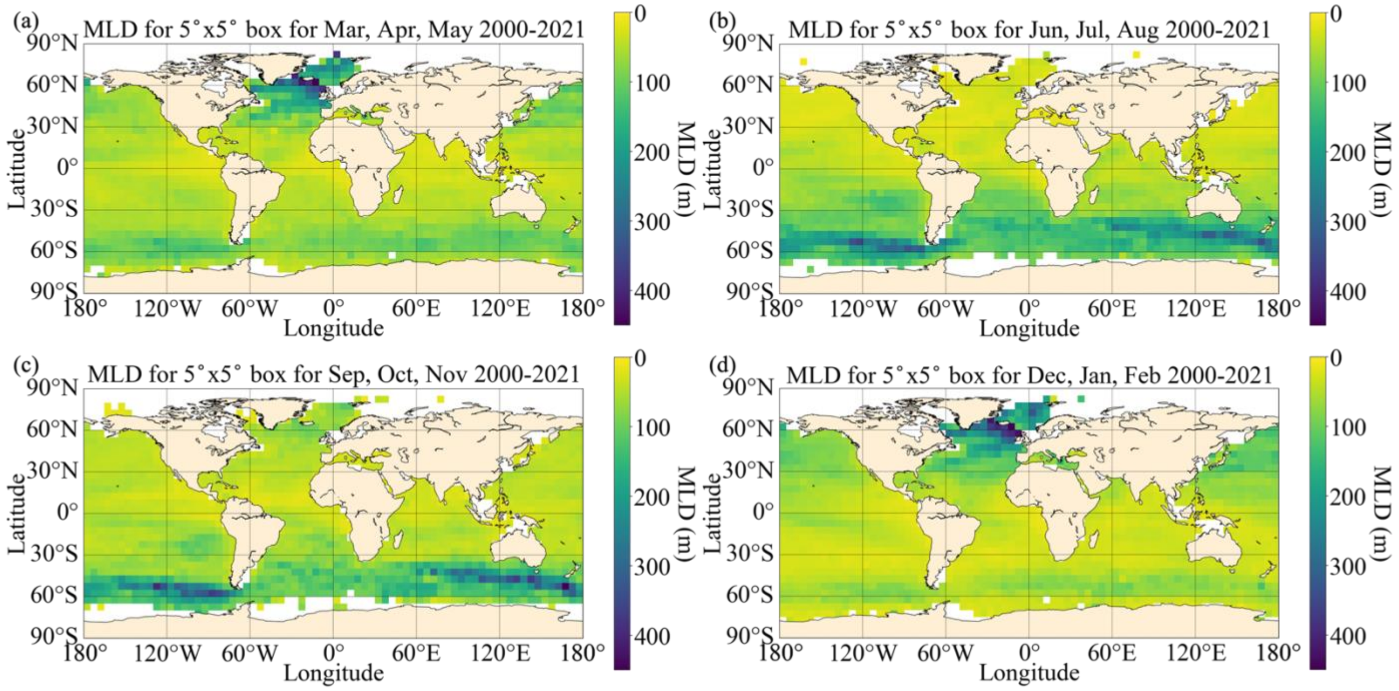

Supplementary Figure 1. Maps of mean mixed layer depth (MLD) in each 5° by 5° longitude-latitude box for (a) March, April, May, (b) June, July, August, (c) September, October, November, and (d) December, January, February for years 2000-2021

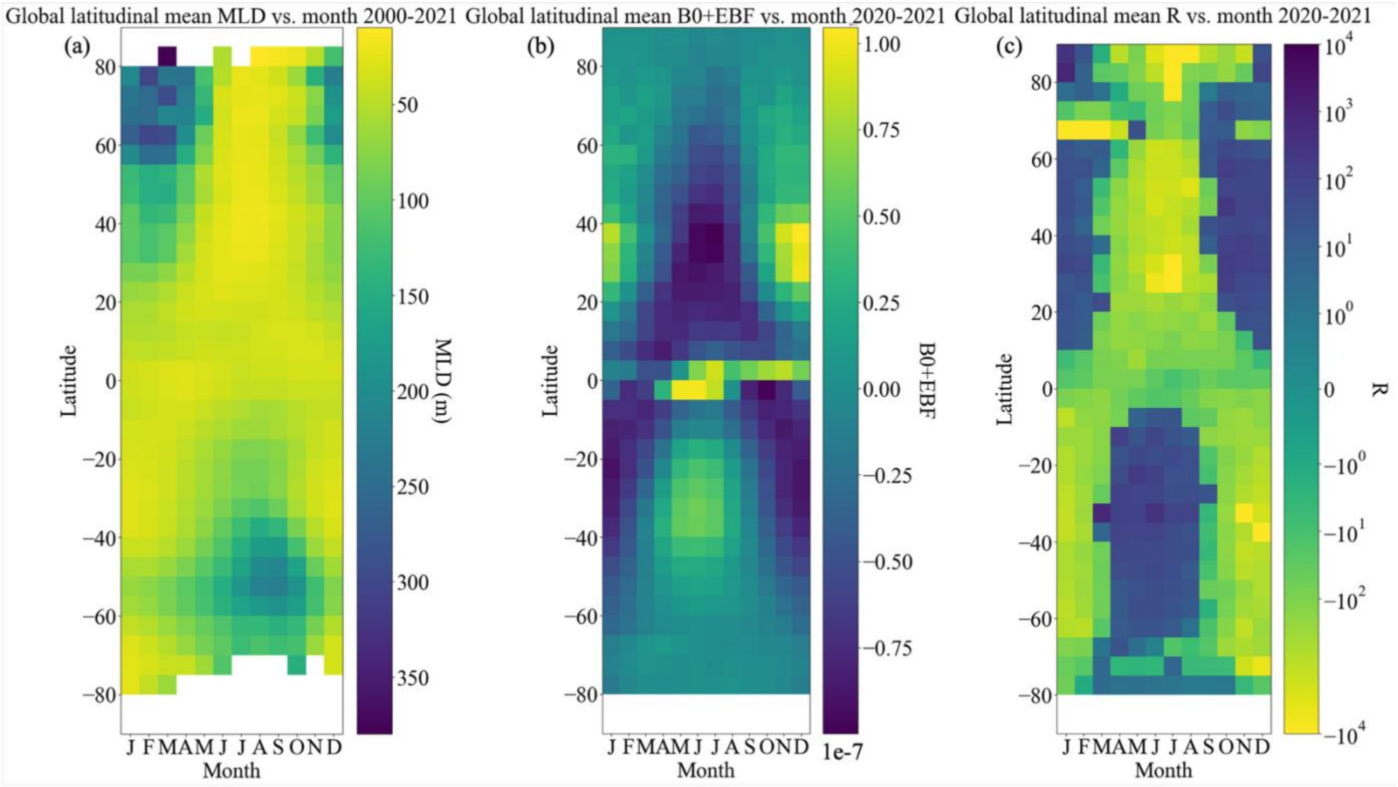

Supplementary Figure 2. (a) Heatmap of global latitudinal mean mixed layer depth (MLD) for each month for years 2000-2021, (b) heatmap of global latitudinal mean surface buoyancy flux due to heat and freshwater air-sea fluxes and Ekman transport ($B_0 + EBF$) for each month for years 2020-2021, and (c) heatmap of global latitudinal mean restratification ratio (R) for each month for years 2020-2021

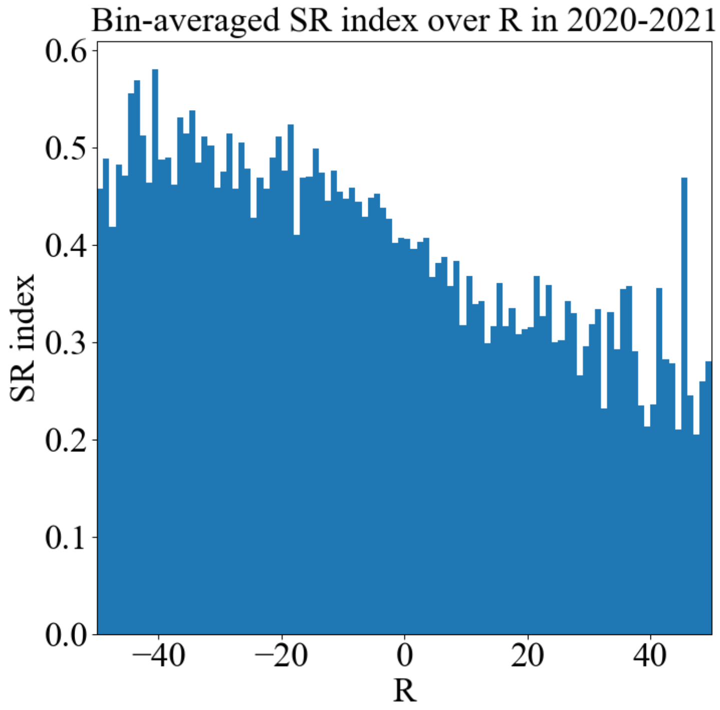

Supplementary Figure 3. Bin-averaged SR index over restratification ratio (R) for years 2020-2021

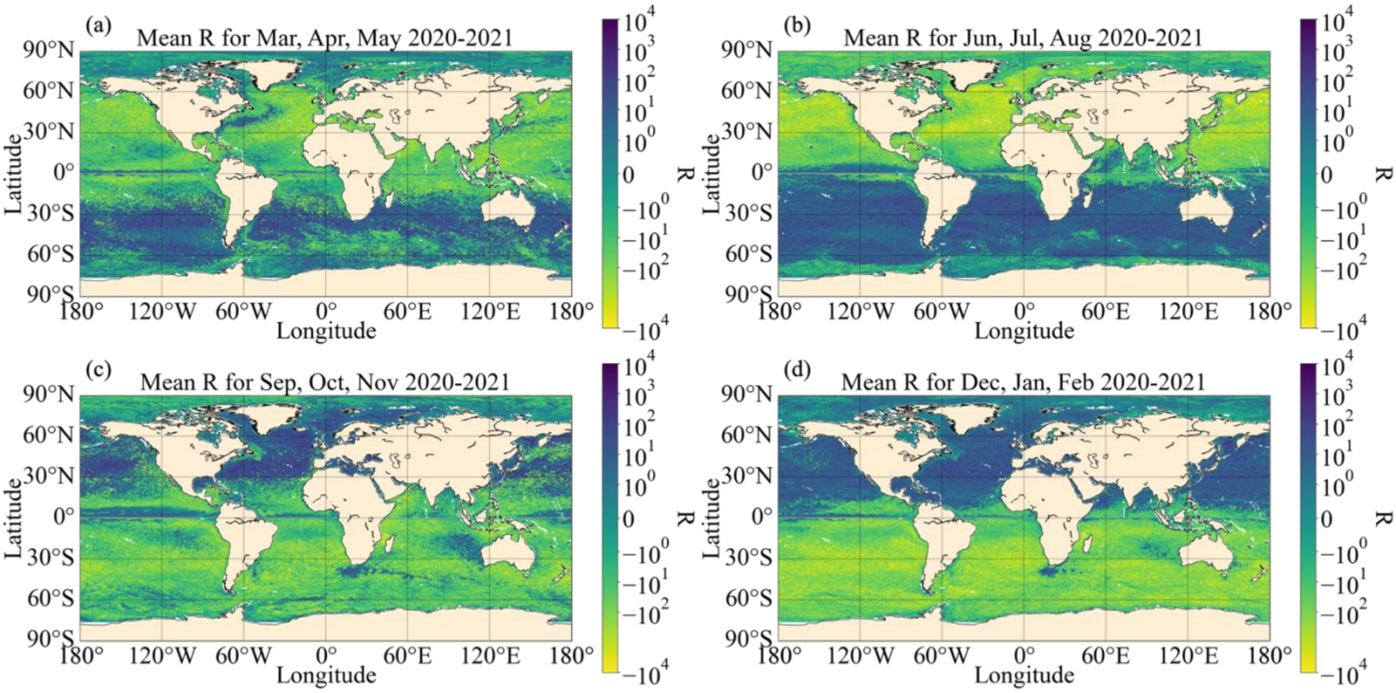

Supplementary Figure 4. Global maps of mean restratification ratio (R) for (a) March, April, May, (b) June, July, August, (c) September, October, November, and (d) December, January, February for years 2020-2021

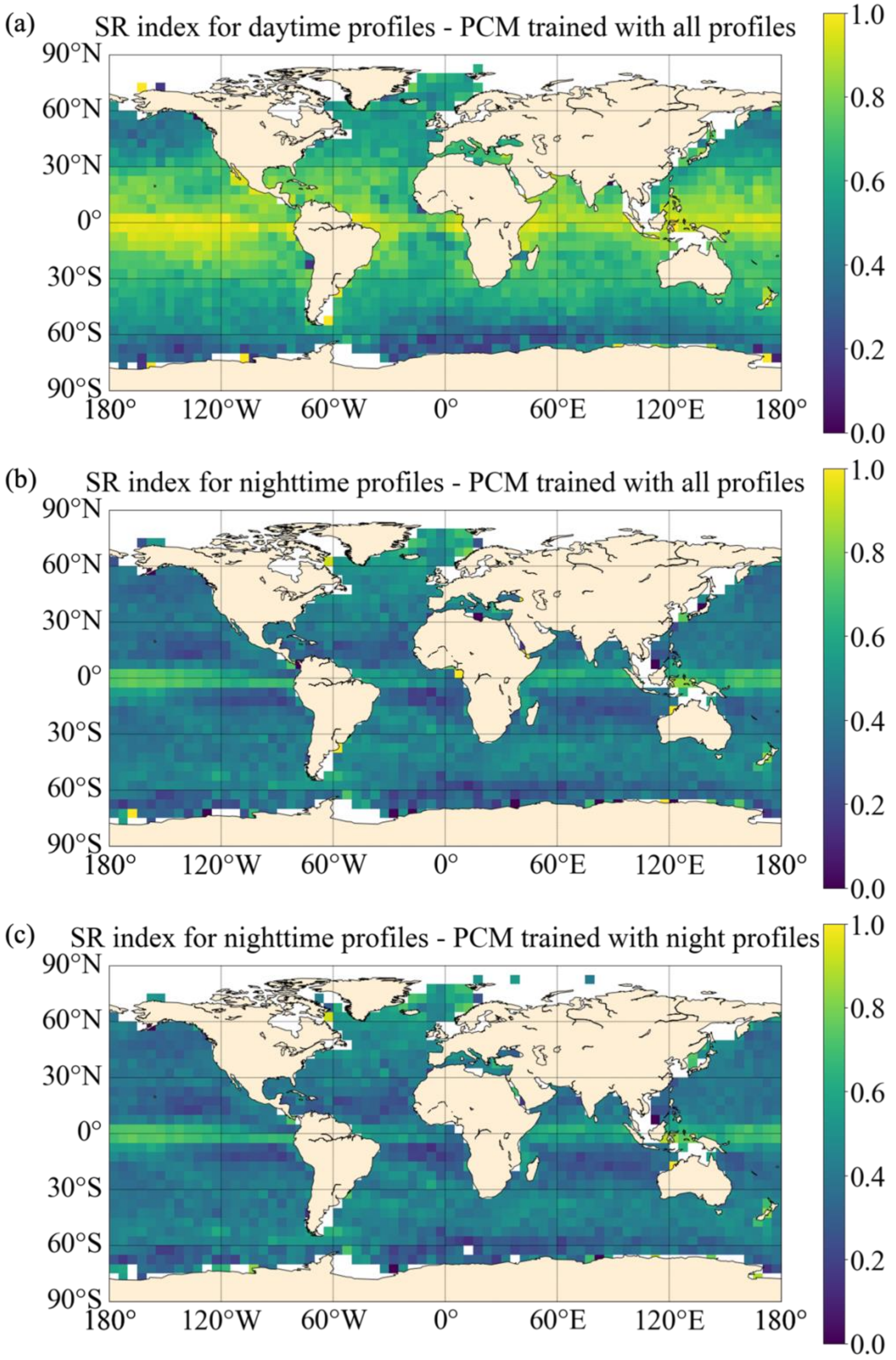

Supplementary Figure 5. Global maps of SR index in each 5° by 5° longitude-latitude box for (a) adapted PCM trained with Argo profiles from all time applied to profiles collected at local day time (10:00-18:00), (b) adapted PCM trained with Argo profiles from all time applied to profiles collected at local night time (22:00-6:00), and (c) adapted PCM trained with Argo profiles collected at local night time applied to profiles collected at local night time

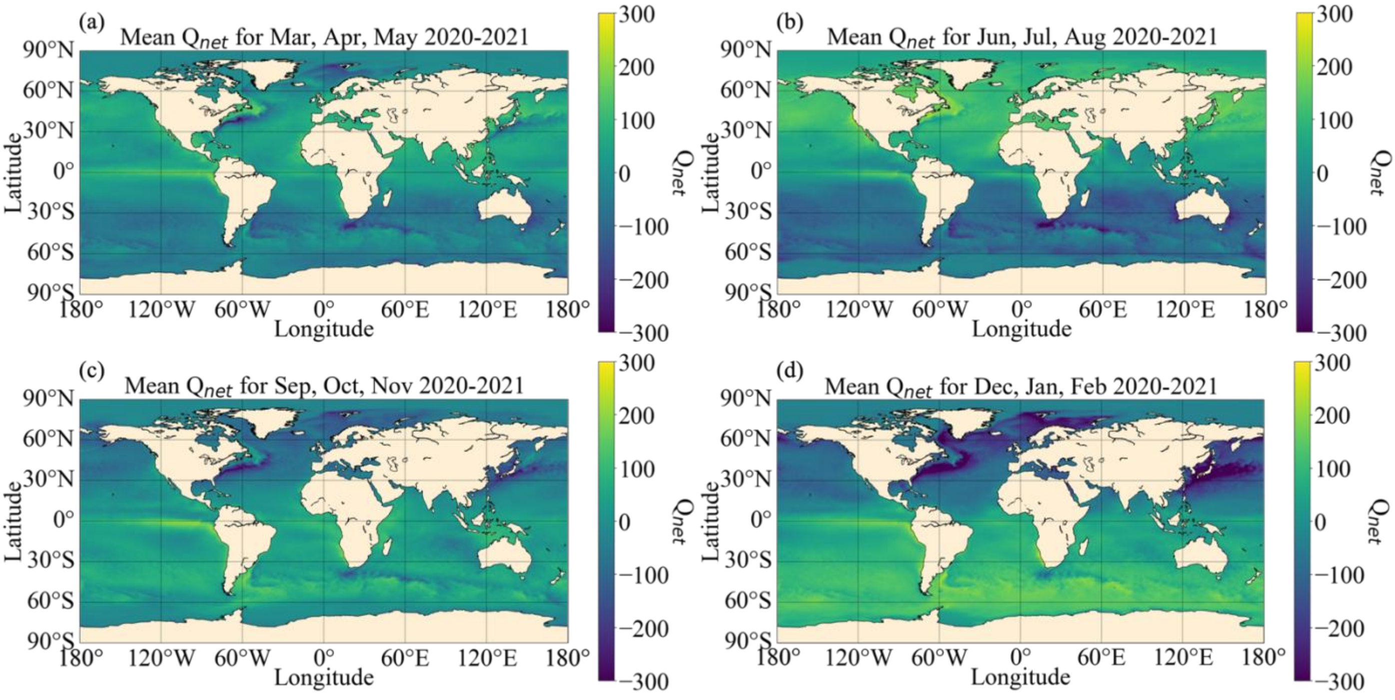

Supplementary Figure 6. Global maps of mean net surface heat flux (Q_{net}) for (a) March, April, May, (b) June, July, August, (c) September, October, November, and (d) December, January, February for years 2020-2021

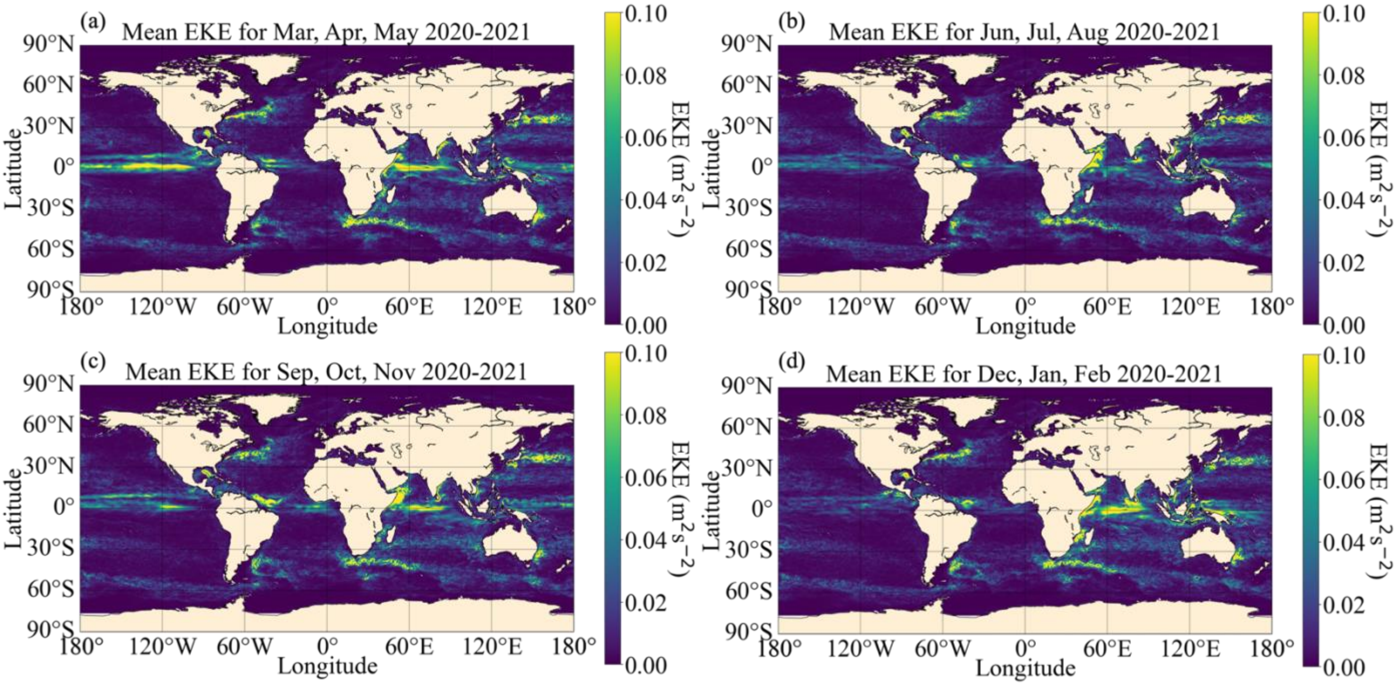

Supplementary Figure 7. Global maps of mean eddy kinetic energy (EKE) for (a) March, April, May, (b) June, July, August, (c) September, October, November, and (d) December, January, February for years 2020-2021

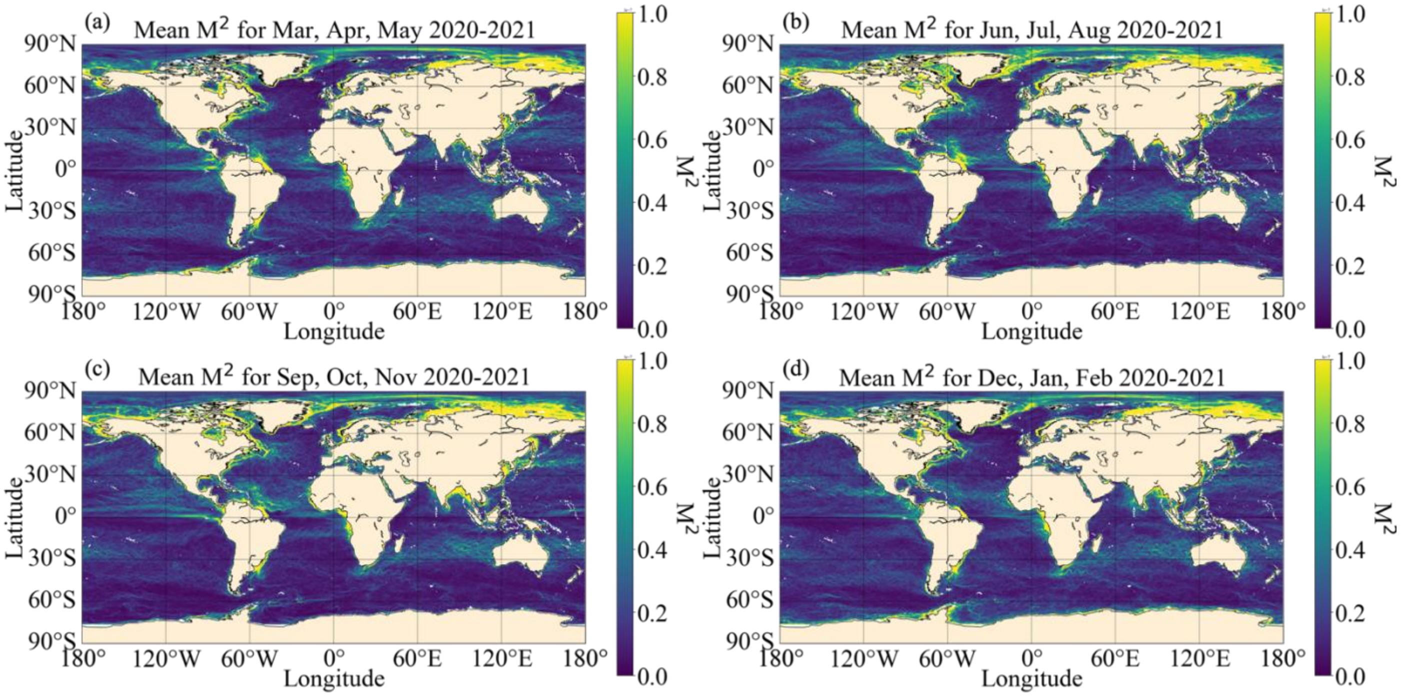

Supplementary Figure 8. Global maps of mean magnitude of ocean surface horizontal buoyancy gradient ($M^2 = |\nabla_h b|$) for (a) March, April, May, (b) June, July, August, (c) September, October, November, and (d) December, January, February for years 2020-2021

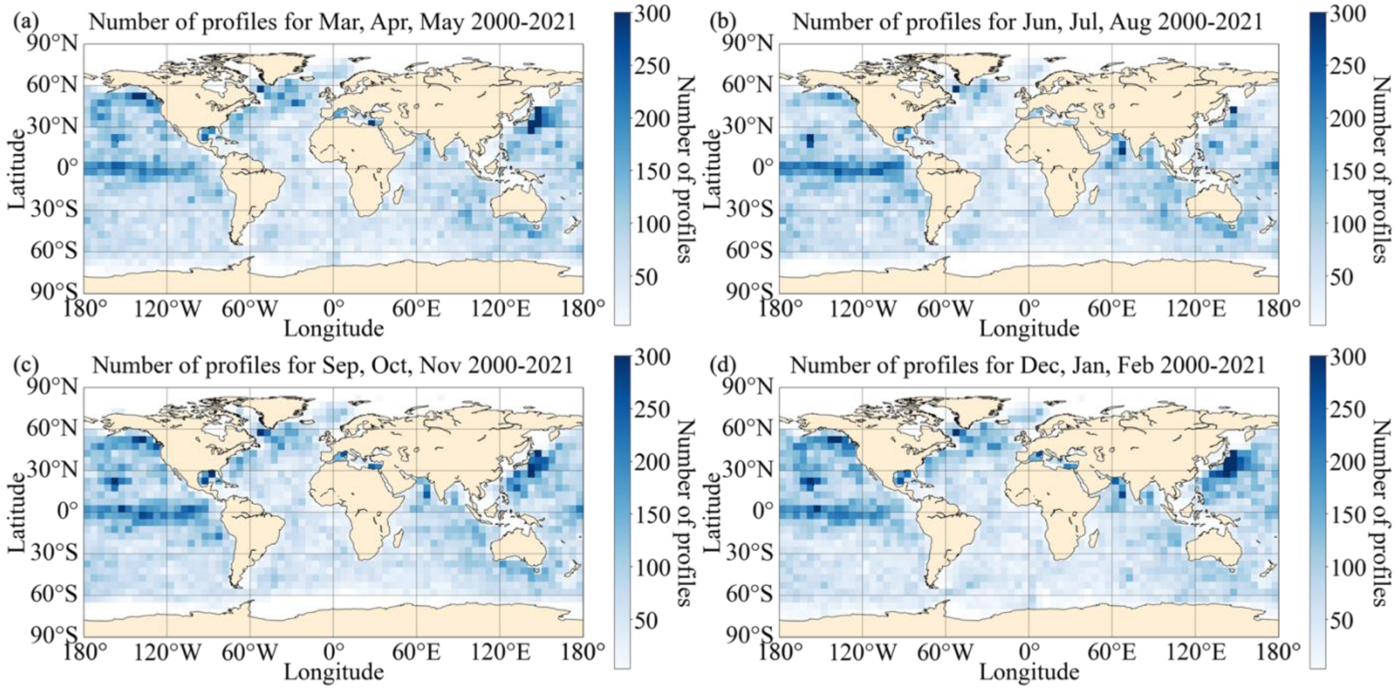

Supplementary Figure 9. Number of total Argo density profiles collected in each 5° by 5° longitude-latitude box (excluding ones with fewer than 3 profiles) for (a) March, April, May, (b) June, July, August, (c) September, October, November, and (d) December, January, February for years 2020-2021

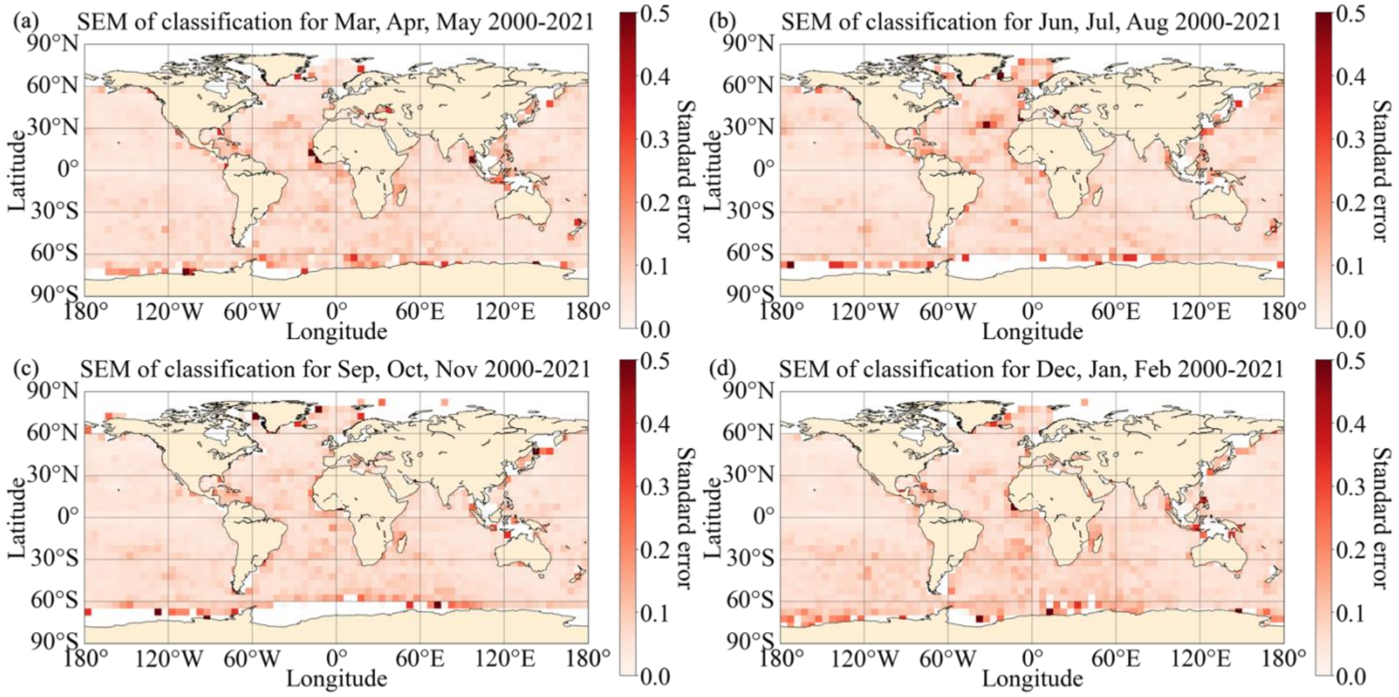

Supplementary Figure 10. Standard error of the mean (SEM) for PCM classification results in each 5° by 5° longitude-latitude box for (a) March, April, May, (b) June, July, August, (c) September, October, November, and (d) December, January, February for years 2020-2021
